# Practical and clinical considerations in Cobalt-60 tomotherapy

**DOI:** 10.4103/0971-6203.54847

**Published:** 2009

**Authors:** Chandra P. Joshi, Sandeep Dhanesar, Johnson Darko, Andrew Kerr, P. B. Vidyasagar, L. John Schreiner

**Affiliations:** 1 Department of Medical Physics, Cancer Centre of Southeastern Ontario (CCSEO), Kingston, ON, Canada; 2 Departments of Oncology and Physics, Queen's University, Kingston, ON, Canada; 3 Department of Physics, University of Pune, Pune, Maharashtra, India

**Keywords:** Cobalt-60, tomotherapy, EGSnrc, dose distribution, head and neck, prostat

## Abstract

Cobalt-60 (Co-60) based radiation therapy continues to play a significant role in not only developing countries, where access to radiation therapy is extremely limited, but also in industrialized countries. Howver, technology has to be developed to accommodate modern techniques, including image guided and adaptive radiation therapy (IGART). In this paper we describe some of the practical and clinical considerations for Co-60 based tomotherapy by comparing Co-60 and 6 MV linac-based tomotherapy plans for a head and neck (HandN) cancer and a prostate cancer case. The tomotherapy IMRT plans were obtained by modeling a MIMiC binary multi-leaf collimator attached to a Theratron-780c Co-60 unit and a 6 MV linear accelerator (CL2100EX). The EGSnrc/BEAMnrc Monte Carlo (MC) code was used for the modeling of the treatment units with the MIMiC collimator and EGSnrc/DOSXYZnrc code was used for beamlet dose data. An in-house inverse treatment planning program was then used to generate optimized tomotherapy dose distributions for the H and N and prostate cases. The dose distributions, cumulative dose area histograms (DAHs) and dose difference maps were used to evaluate and compare Co-60 and 6 MV based tomotherapy plans. A quantitative analysis of the dose distributions and dose-volume histograms shows that both Co-60 and 6 MV plans achieve the plan objectives for the targets (CTV and nodes) and OARs (spinal cord in HandN case, and rectum in prostate case).

## Introduction

Cobalt-60 (Co-60) teletherapy was the established high energy radiation treatment of cancer in the 1950s. In the past five decades, tremendous research and innovation has gone into improvement of radiation therapy including implementation of three dimensional conformal radiation therapy (3DCRT) and intensity modulated radiation therapy (IMRT) techniques. Most of these advances have been using medical linear accelerators (linacs) and modernization in Co-60 based external beam radiation therapy (EBRT) units has been almost negligible. For example, the Co-60 unit is yet to be equipped with a multi-leaf collimator, which is an essential element of efficient delivery of conventional 3DCRT and a basic requirement for the implementation of IMRT techniques.

The lack of significant development in Co-60 EBRT technology may be attributed to some perceived disadvantages such as relatively lower photon energy (average 1.25 MeV), lower radiation output and larger beam penumbra associated with Co-60 radiation.[1–2] However, a number of studies have pointed out that if the Co-60 unit is modernized, with state of the art devices, these issues would have an insignificant impact on the overall dose distribution.[3–8] Johns and Cunningham (1983) demonstrated that differences in the dose distribution achieved in bladder treatment with 10 MV x-rays and with Co-60 γ-rays become minor when rotational approaches are used.[6] Other researchers have also reported that with advanced multi-beam treatment modalities, the advantage of treatment with very high-energy photons (greater than10 MV linacs) decrease and become negligible, particularly when IMRT or tomotherapy techniques are used.[6–8] Furthermore, Joshi *et al*.(2001, 2008) noted that the problem of low dose rate for fan beam applications, for a given source strength can be improved if the Co-60 unit is redesigned to include multiple sources, decreased source to axis distance (SAD) and/or using a different source shape and packing density.[9,10] Considering these solutions to be preceived problems, along with the robustness of the unit in terms of maintenance, safety and cost, put Co-60 in a better light with respect to linac based radiation therapy than held by conventional wisdom.[1–3,11] It has been suggested that Co-60 could have an increased role in radiation therapy if some new thought went into the design of improved clinical units.[1–4]

The Medical Physics research group at the Cancer Centre of Southeastern Ontario (CCSEO) and Queen's University has proposed a Co-60-based tomotherapy as a form of treatment delivery. Tomotherapy employs a radiation source revolving around the patient with a collimated narrow radiation fan-beam that projects a slice onto the patient. The radiation intensity across each slice is modulated using a digitally controlled binary multi-leaf collimator (BMLC). The dose delivery can be achieved using two approaches, axial and helical. In axial tomotherapy, one slice is treated per rotation of the intensity modulated radiation fan beam and the patient is translated in steps. This is implemented, for example, in linacs with the binary ‘multi-leaf intensity-modulation collimator’ MIMiC® (NOMOS Corporation, Sewickly, PA).[12] In helical tomotherapy, the radiation source is mounted on CT-like gantry and the conformal dose delivery is achieved by the continuous translational motion of the patient through the gantry simultaneous with the intensity modulated radiation fan beam rotations. Currently, the Hi-Art tomotherapy unit (Tomotherapy Inc., Madison, WI), using a 6 MV linac x-ray source, is the only commercially available helical tomotherapy unit.[6,13]

Feasibility studies have demonstrated that Cobalt-60 tomotherapy is viable.[4,9,14–15] In this work we will report on the results of modeling investigations of Co-60 based tomotherapy. In particular, two clinical cases of head and neck (HandN) and prostate cancer are studied and the Co-60 tomotherapy dose distributions compared to those obtained with 6 MV linac-based tomotherapy. The treatment of head and neck (HandN) cancer using conventional three-dimensional (3D) conformal radiation therapy (3DCRT) techniques is often very challenging due to location of various malignant target structures (e.g. gross tumour volume (GTV), clinical target volume (CTV) and involved lymph nodes, etc.) close to critical normal structures (e.g. the eyes, spinal cord, optic chiasm, parotid etc.). The treatment of the prostate, located in the pelvic region, provides an anatomical site with a larger separation.

## Materials and Methods

Tomotherapy dose distributions were generated on a single H and N and prostate computed tomography (CT) slice with a clinical T780c Co-60 unit (with a 2 cm diameter cylindrical source) and a 6 MV photon beam from a linac (CL2100EX, Varian Medical systems, Palo Alto, USA). Both these units were modelled with the standard retrofit MIMiC MLC system (NOMOS Corp., Swickley, PA). The T780c Co-60 unit and the 6 MV linac were modelled with collimator to isocentre distances (CIDs) of 30 cm and 38.85 cm, respectively; and with source to axis distances (SADs) of 80 cm and 100 cm, respectively. These dimensions correspond to the actual distances in these units when retrofit with the MIMiC MLC system.

Monte Carlo (MC) simulations were used to calculate the percentage depth dose and profile data for pencil beams (or beamlets) that could be then combined to produce fan beam intensity modulation. The BEAMnrc and DOSXYZnrc Monte Carlo codes were used for beamlet dose profile calculations.[17–18] The BEAMnrc MC code was used for modelling the treatment units and calculation of the phase-space data for beamlets obtained from the MIMiC collimator. The CT slices from the patient image data for the two sites studied were converted into a phantom model for calculation of the dose distributions The DOSXYZnrc MC code, utilizing the BEAMnrc phase-space data as input, was used to calculate the doses in the desired phantom for the individual leaf pairs and for each of the treatment units. Tomotherapy plans were then generated for a set number of pre-defined beam orientations and optimized for a set of pre-defined objectives with an in-house inverse treatment planning program.[18,19] The target structures in the HandN case were clinical target volume (CTV) and left and right posterior neck nodes; the organs at risk (OAR) structures were the spinal cord and the remaining normal tissue. In the prostate case, the prostate was outlined as the CTV while the rectum was outlined as an OAR.

In the H and N case, the dose distributions for both Co-60 and 6 MV were calculated in a homogeneous, water-equivalent material (i.e. with heterogeneities not considered). 16 individual beamlets from the MIMiC collimator were used to obtain a fan beam modulation and then the fan beams were individually simulated for 51 equally spaced gantry angles. A matrix resolution of 2×2 mm^2^ was used for the dose calculations. A dose of 70 Gy to CTV was prescribed to treat the primary cancer while doses of 66 Gy to the right posterior neck node and 50 Gy to the left posterior neck node were considered adequate for treatment of the nodal disease. A variation of plus/minus five per cent between the prescribed and delivered doses in the CTV and the nodal regions was considered acceptable in the optimization. The dose to the spinal cord, an organ at risk (OAR), was limited to a maximum of 40 Gy. Both Co-60 and 6 MV plans were analyzed by comparing the isodose distribution, the dose difference map, and dose area histograms (DAHs), which are, two-dimensional versions of the conventional dose volume histograms (DVHs).

In the prostate case, the dose distributions were calculated taking into account all heterogeneities. In this case, because the maximum dimension of the prostate volume was equal to or less than 8 cm, only the central 10 out of the 20 leaves of the MIMiC were used and the rest of the leaves were kept closed at all times. These beamlets were simulated individually for 21 equally spaced gantry angles. A dose of 76 Gy was prescribed to the prostate. Only a portion (20%) of the rectum was allowed to receive a maximum dose of 70 Gy. The plan was considered acceptable if the dose to the prostate was between 95 - 105% of the prescribed dose. Both the prostate and rectum were assigned higher importance factors to ensure that the prostate receives the prescribed dose and to minimize the dose to the rectum.

## Results and Discussion

### Comparisons of Head and Neck Co-60 and 6 MV Tomotherapy Plans

**Figure 1:**
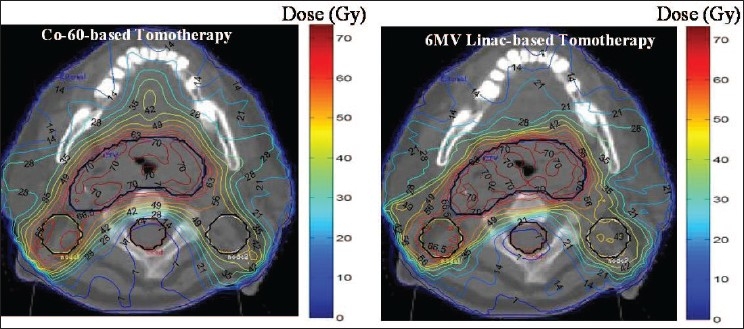
A review of the optimized HandN tomoptherapy plans for a 6 MV linac and the conventional T780c Co-60 unit incorporating the MIMiC binary multi-leaf collimator. The left figure shows the dose distribution for a T780c delivery and the right figure shows the distributions for a 6 MV linac delivery

Figure 1 presents dose distributions of tomotherapy plans for the treatment of a tumor located in the the HandN region by the 6 MV and T780c units modeled with a retrofitted MIMiC multi-leaf collimator. Both units are able to deliver highly conformal treatment doses around the target structures and low doses in the OAR and the normal tissue regions. The 6 MV and T780c plans deliver the maximum spinal cord doses of 14 Gy and 13.7 Gy, respectively. Both plans deposit a maximum dose of 68.5 Gy to the normal tissue, whereas the average dose in the same region were 26 Gy and 27.5 Gy for the 6 MV and the T780c plans, respectively. In both plans, the maximum doses to the cord were significantly lower than the 40 Gy maximum point dose criteria set for the spinal cord. Thus, for both of the avoidance structures (i.e. spinal cord and normal tissue), 6 MV and T780c tomotherapy plans showed similar doses.

**Figure 2:**
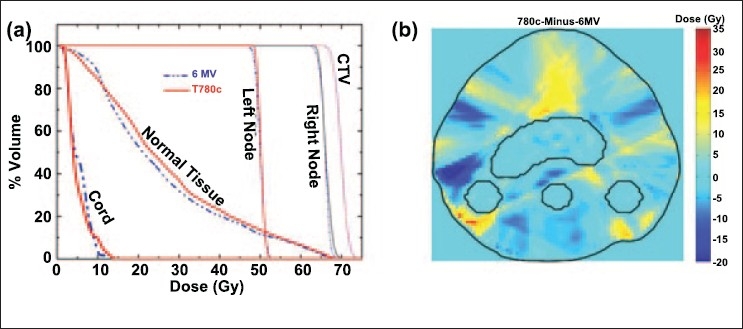
(a) The dose area histogram (DAH) for the HandN tomotherapy plans from 6 MV linac and T780c Co-60 units with MIMiC MLCs. (b) The dose difference map: T780c plan minus 6 MV plan. The red and yellow regions illustrated higher doses (in Gy) with the T780c plan, the green and blue regions represent the higher doses with the 6 MV plan and the regions with background colour represent the region with negligible dose difference between the two plans

These plans are further compared using the DAHs and the dose difference map in Figure 2. A comparison of the DAHs further emphasizes the similarity of these plans for all volumes of interest [Figure 2a], where a very small differences in the doses in the spinal cord and normal tissue is indicated. The dose difference map shows the regions of dose differences between the plans [Figure 2b] with the yellow regions indicating the higher doses, and green and blue showing lower doses delivered with the T780c plan. The dose difference map shows similar regions of high and low doses between the plans.

### Comparisons of Prostate Co-60 and 6 MV Tomotherapy Plans

**Figure 3:**
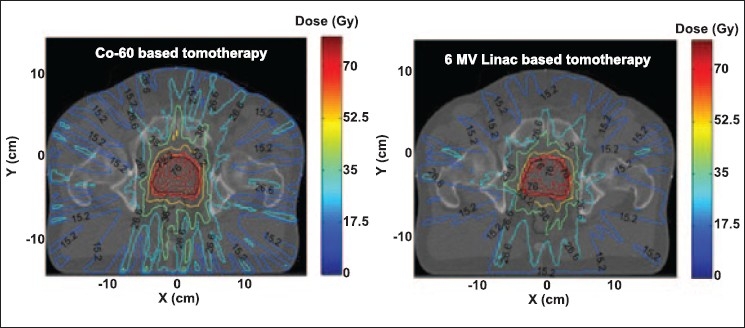
Tomotherapy plans for Co-60 and 6 MV beams for a prostate case, achieved by modulating the intensity of 210 beamlets (10 beamlets per orientation and 21 equally spaced field orientations)

Figure 3 shows the conformal dose distributions for a prostate case generated using Co-60 and 6 MV linac based tomotherapy. As desired, the high dose treatment for both tomotherapy plans is concentrated within the prostate and only a small portion of the rectum receives a high dose. The dose to the external body is also limited. These results prove that it is possible to provide a highly conformal radiation therapy via Co-60 based tomotherapy when treating deep seated tumor situated in anatomical regions with large separations such as the pelvis.

**Figure 4:**
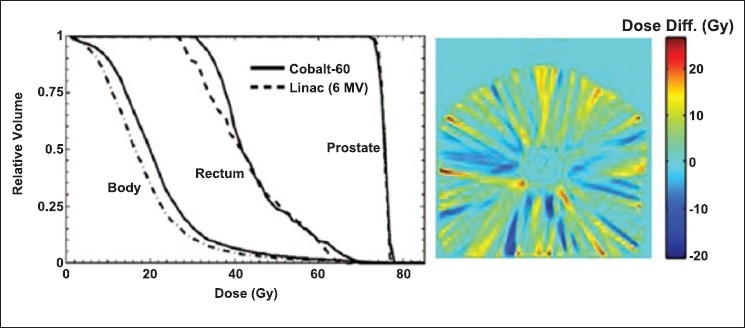
Left: Cumulative dose area histograms for Co-60 and 6 MV linac based tomotherapy. Right: Dose difference map of Co-60 plan and 6 MV linac plan. In the dose difference map the yellow and red represent the regions where Co-60 is giving more dose than 6 MV and the blue regions represent the regions where 6 MV is giving more dose

The cumulative dose area histograms in Figure 4, representing a quantitative analysis of the plans, show that both Co-60 and 6 MV linac based tomotherapy give similar dose to the prostate. Although in the Co-60 plan the dose to the body and rectum is slightly more in low dose regions than the 6 MV plan, the dose to these regions is within the clinically acceptable dose limits. This dose difference depends on beam energy, total number of beams as well as the optimization parameters. The difference will potentially become smaller if total number of beams is increased. In this study only 21 gantry angles were used while a typical pelvic tomotherapy treatment is likely to employ higher number of gantry angles. The dose difference map in Figure 4 shows the regions that receive relatively more or less dose when compared to that in the 6 MV plan. The positive numbers represent the regions where the dose in the Co-60 plan is higher and the negative numbers represent the regions where the dose in the 6 MV plan is higher.

## Conclusions

This work shows that Co-60 tomotherapy is clinically viable. The detailed comparison and analysis of tomotherapy dose distributions for HandN and prostate cases demonstrate that Co-60 tomotherapy can provide dosimetrically competitive solutions compared to the 6 MV linac based tomotherapy. We have also shown that contrary to the perceived believed that Co-60 is limited by its relatively lower energy, comparable and clinically acceptable tomotherapy plans are possible for the treatment of cancers located in smaller (e.g. HandN) as well as larger (e.g. prostate) anatomical regions. Further research is underway to extend these studies to three dimensions.
